# Advancements in Laser Therapies for Dermal Hyperpigmentation in Skin of Color: A Comprehensive Literature Review and Experience of Sequential Laser Treatments in a Cohort of 122 Indian Patients

**DOI:** 10.3390/jcm13072116

**Published:** 2024-04-05

**Authors:** Suruchi Garg, Kanya Rani Vashisht, Diksha Garg, Bhavni Oberoi, Geeta Sharma

**Affiliations:** 1 Aura Skin Institute, Chandigarh 160009, India; drdiksha.garg@gmail.com (D.G.); geetasharma39015@gmail.com (G.S.); 2 Postgraduate Institute of Medical Education and Research (PGIMER), Chandigarh 160012, India; kanyavashisht@gmail.com; 3 INHS Asvini, Mumbai 400005, India; bhavni.oberoi@gmail.com

**Keywords:** dermal hyperpigmentation, skin of color, Q-switched lasers, picosecond lasers, skin pigmentation, dermal melanosis, acquired dermal macular hyperpigmentation, dermal melanocytosis, tattooing, nevus of Ota

## Abstract

The heightened awareness of ethnic dermatology aligns with the growing prevalence of skin of color communities globally, where hyperpigmentation disorders pose a common dermatological challenge. Effectively addressing dermal pigmentation is challenging due to its resistance to conventional therapies and its association with impaired quality of life. This underscores the need for effective treatments and a thorough grasp of laser advancements. A relevant literature search spanning the last 7 years across the PubMed database reveals core studies, challenges, and the evolution of laser technologies tailored for various forms of congenital and acquired dermal hyperpigmentation in skin of color. This comprehensive review explores the mechanisms, applications, and recommendations for pigmentary laser technologies, highlighting the key role of Q-switched lasers in their established millisecond/ nanosecond forms and emerging picosecond lasers, fractional non-ablative and ablative lasers, Intense Pulsed Light, etc. The summary of evidence includes studies on dermal melanocytosis (nevus of Ota and Hori’s nevus), tattoos, acquired dermal macular hyperpigmentation, etc., and also entities with mixed epidermal–dermal components, such as melasma and post-inflammatory hyperpigmentation. The review offers valuable insights for clinicians to make informed decisions based on diagnosis, skin type, and the latest technologies to optimize results and minimize complications, especially in darker Fitzpatrick skin types. In their five-year study with 122 Indian patients, the authors applied specific laser combinations for diverse dermal melanoses, including tattoos, dermal/mixed melasma, acquired dermal macular hyperpigmentation, and dermal nevi. Substantial pigmentation reduction, subjectively assessed by both physicians and patients, was observed across all groups. A one-way ANOVA indicated a significant difference in mean improvement scores across various pigmentary conditions (F = 3.39, *p* = 0.02), with melasma patients exhibiting a significantly higher improvement score than tattoos (*p* = 0.03). The results affirmed the safety and efficacy of sequential laser therapy for dermal pigmentation in skin of color, advocating for flexibility in approach while maintaining the rationale behind the laser sequences. Despite advancements, challenges persist, and gaps in the current literature are identified. In conclusion, this summary highlights the ongoing pursuit of optimal protocols in dermatological laser treatments for dermal melanoses, offering valuable insights for future research and clinical practice.

## 1. Introduction

The growing acknowledgment of ethnic dermatology aligns with the rising prevalence of skin of color (SoC) communities globally. While ethnic skin exhibits a lower incidence of skin cancer due to the photoprotective role of melanin, diseases of cosmetic concern, including pigmentation disorders, are more prevalent in these populations. In fact, they are among the commonest dermatological concerns affecting SoC, causing significant psychological and social distress found in clinical practice, especially when exposed sites are affected [1,2,3,4]. Understanding the specific challenges and characteristics of pigmentation disorders in this context is crucial for developing effective and inclusive dermatological interventions. While epidermal lesions like freckles and lentigines show a predictable response to treatment, addressing dermal pigmentation poses difficulty, particularly in SoC, in terms of a higher risk of adverse events and inadequate response to conventional treatments. Certain laser systems, however, are deemed effective due to their precision and depth of penetration while relatively sparing the epidermis, provided the correct technology and parameters are chosen [1,3]. This comprehensive review explores the latest laser therapies tailored for dermal hyperpigmentation over the last 7 years, with a focus on ethnic skin. It delves into various cutting-edge laser technologies, emphasizing their progress, evolution, and applications in addressing dermal pigmentation disorders, including tattoos, dermal nevi (nevus of Ota), dermal/mixed melasma, acquired dermal macular hyperpigmentation, etc. A multifaceted understanding of the subject is provided, synthesizing data from a meticulous literature search in the PubMed database, along with insights gained from the authors’ experiences with 122 Indian patients.

### 1.1. An Overview of Entities

Skin hyperpigmentation disorders are categorized into epidermal, dermal, or mixed epidermal–dermal, based on the location of pigment deposition. This review deals with the various forms of dermal pigmentation (Table 1) and primarily excludes drug-induced, mucosal, and nail pigmentation, as they fall beyond its scope [5]. Epidermal hyperpigmentation is usually light-to-dark brown, while dermal hyperpigmentation may display blue–gray tones attributed to the Tyndall effect, where short wavelengths of blue light scatter more than longer wavelengths. While the former arises from an increase in the deposition of melanin or the number of melanocytes, the latter results from the presence of melanophages, melanin, or hemosiderin due to damage to basal keratinocytes by various insults. While Wood’s lamp has been used historically to determine pigment location, recent histopathologic studies question its accuracy, and dermoscopy may be a valuable alternative [5]. Some hyperpigmentation types, like melasma and post-inflammatory hyperpigmentation (PIH), may present a mixed pattern involving both epidermal and dermal components, challenging rigid categorization based on pigment depth [6]. In exploring human skin color variations, recent literature highlights the role of dermis-derived fibroblast factors in regulating skin pigmentation [7]. These considerations are relevant for accurate diagnosis and targeted treatment of various hyperpigmentation disorders.

### 1.2. Effects on Quality of Life

Dermal pigmentation disorders, particularly when affecting the face and neck, can have a substantial impact on patients’ quality of life, given their chronic and treatment-resistant nature. A study noted individuals with pigmentary dermatoses were significantly more depressed than those with other facial skin conditions such as acne, acne scars, and rosacea [8]. An assessment using the Dermatology Life Quality Index (DLQI) revealed substantial impacts on quality of life, with 42% and 40% of patients experiencing a “very large” or “moderate” effect in cases of Riehl’s melanosis and lichen planus pigmentosus, respectively [9]. In fact, the psychosocial burden of the latter was comparable to vitiligo, affecting domains such as feelings and social interaction [10]. A population-based survey in France revealed that tattooing was associated with lower quality of life scores, particularly among individuals with multiple tattoos [11]. Psychological disturbances, including anxiety and depression, may ensue, thus emphasizing the need for future research for more effective therapy [12].

### 1.3. Challenges in Treating Dermal Pigmentation

Effectively addressing dermal pigmentation is notably challenging, especially in SoC, given its resistance to medical interventions, chronic nature, heightened risk of PIH following therapy, and the requirement for more treatment sessions on average compared to epidermal pigmentation [13]. Treatment outcomes are often unpredictable, and pigmentation recurrence is common. With these concerns, it is not surprising that there is a lack of confidence and inconsistent management practices among dermatologists when treating such disorders [1,14]. Moreover, challenges in evaluating individuals with SoC arise due to the variability between races and ethnicities, and the Fitzpatrick scale, used to assess skin phototype, is noted to be insufficient in fully characterizing the diversity within these populations [2,15]. Case selection gains prominence since non-indicated laser treatment of skin lesions may impede the diagnosis of malignant skin lesions [16]. Laser parameters are typically determined based on pigment levels, among other factors. However, conditions such as melasma and PIH cannot be strictly compartmentalized into epidermal or dermal categories based on pigment depth, and conducting histological studies may not be practical [6]. As the cosmetic community shows a growing interest in using lasers to address these issues, it becomes imperative for physicians administering laser treatments to possess an adequate understanding of the subject and its advancements over recent years [13]. With this background, a comprehensive literature review becomes valuable for seeking evidence to guide treatment decisions based on the diagnosis and skin type.

### 1.4. Mechanisms and Evolution of Pigmentary Laser Systems

Dermatology has extensively utilized laser treatment, wherein the energy of photons is determined by the wavelength, pulse duration (PD) determines the time of energy delivery to the tissue, and thermal relaxation time (TRT) indicates the time required for the heated target tissue to dissipate 50% of the absorbed energy via thermal diffusion. Laser–tissue interactions include photothermolysis (causing tissue vaporization or melting), photomechanical/photoacoustic effects (generating an acoustic shockwave), photochemistry (triggering chemical reactions, breaking bonds and tissue weakening), and photobiomodulation (altering cellular signaling pathways). “Selective photothermolysis” occurs when PD is shorter than TRT, resulting in specific thermal damage to the target tissue [17]. Various lasers are employed for treating pigmented lesions, relying on this principle. Optimal wavelengths target pigments, particularly melanin, with minimal absorption by hemoglobin or water. To achieve laser selectivity, PD should be much shorter than the TRT of the target (melanosomes), often below 100 ns [18].

Q-switched (QS) lasers produce short pulses of high-intensity beams and are considered the “gold standard” for treating pigmented lesions [6,13]. Lasers with longer wavelengths, like the 1064 nm QS neodymium: yttrium aluminum garnet (Nd:YAG) laser, can penetrate the dermis more effectively, making them a preferred choice for addressing dermal pigmentation, particularly in dark skin [19]. It targets melanosomes in melanocytes, keratinocytes, and melanophages with its short pulse width and adjustable spot size [3]. The large spot size of up to 10 mm enables deep tissue penetration, and a “top-hat” beam profile ensures uniform energy distribution, with some systems offering a fractional handpiece for treating rejuvenation and pigmentary conditions like melasma [13]. Other QS lasers with shorter wavelengths (Ruby 694 nm, frequency-doubled Nd:YAG 532 nm) are more suitable for superficial (epidermal) lesions and have a higher risk of PIH. The QS-Alexandrite laser (755 nm) has an intermediate wavelength, allowing its use for both epidermal and dermal pigmented lesions [13]. Intense Pulsed Light (IPL) technology, which encompasses a range of noncoherent wavelengths, is not considered suitable for dermal pigmentation as a standalone therapy [13]. The 511 nm copper bromide and 595 nm pulsed-dye lasers may also be effective.

Laser systems, previously limited to milliseconds for PDs, have now progressed toward technologies with PDs in the nanosecond (ns) and picosecond (ps) range for treating resistant dermal pigmentation [17]. Within the dermal layer, melanosomes absorb brief bursts emitted by the device, providing sufficient energy to break down tissue while minimizing collateral thermal damage. Despite advancements, ns-QS lasers still pose challenges with multiple treatments and instances of dyspigmentation and scarring, attributed to the main mechanism of photothermal damage, an excess of which may trigger inflammation and alteration of melanocytes in the basal epidermis, especially in darker skin tones [20,21]. This prompted the development of a technology with optimal photoacoustic effects for this patient population, viz., the ps-lasers, operating in the sub-nanosecond range (10^−12^ s). Approved by the FDA for skin applications in 2012, ps-lasers, characterized by extremely brief PDs and substantial peak power density, rely on photoacoustic destruction rather than photo-thermolysis. The heightened photomechanical/photoacoustic effect and reduced heat diffusion into nearby structures potentially offer improved clinical outcomes and decreased thermal injury compared to ns-domain devices [20]. In terms of wavelength, these include the ps-Alexandrite (755 nm) and ps-Nd:YAG (1064 nm) lasers. Furthermore, when equipped with fractional optical delivery systems such as the Diffractive micro-lens array, lasers produce an array of focused micro-spots featuring increased fluence or concentrated energy. This results in neighboring tissue receiving lower fluence, thus remaining relatively unharmed [17].

## 2. Methods

A comprehensive literature search was conducted across the PubMed database for relevant articles over the last 7 years on 23 December 2023, using keywords (both individually and in combination), including MeSH as well as non-MeSH terms such as “dermal pigmentation”, “dermal melanosis”, “dermal hyperpigmentation”, “tattoo”, “mixed melasma”, “dermal melasma”, “acquired dermal macular hyperpigmentation”, “dermal melanocytosis”, “Nevus of Ota”, “pigmentary lasers”, etc. Articles that were case reports, not in English or not pertaining to SoC, were excluded. The references of pertinent articles were further reviewed to identify additional relevant sources. This review article adheres to ethical standards.

## 3. Discussion

### 3.1. Individual Entities: Summary of Evidence

#### 3.1.1. Dermal Melanocytosis (Nevus of Ota and Hori’s Nevus)

Nevus of Ota is a form of congenital dermal melanocytosis primarily occurring along the trigeminal distribution, prevalent in SoC populations [22]. When bilateral, it is called Hori’s nevus or “acquired bilateral nevus of Ota-like macules”. The condition can be cosmetically disfiguring [23]. Prior to the introduction of the QS lasers, treatment approaches like dermabrasion and cryotherapy often resulted in dyspigmentation or scarring [20]. For nevus of Ota, the 1064 nm QS Nd:YAG laser stands out as the most extensively used treatment modality, offering effective results, especially in higher Fitzpatrick skin types. The treatment, however, requires multiple sessions with intervals of at least two months, with potential side effects like purpura and PIH. Hori’s nevus and blue nevi also respond well to QS laser treatment [13]. Ps- or ns-ranged QS pigment (Nd:YAG, Ruby, Alexandrite) and vascular (KTP or Potassium titanyl phosphate) lasers have all demonstrated effective clinical outcomes and gained broad acceptance as the established standard of care in terms of studies as well as formal recommendations [20]. However, millisecond lasers and IPL have not been found to be effective [16]. A recent study comparing the ps and ns forms of 755nm laser demonstrated superior efficacy and fewer side effects in the former [24]. Younger patients show better responses, requiring fewer sessions and less fluence [25,26]. Potential explanations include nevi darkening, darkening of the surrounding skin, and an increase in dermal melanocytes’ histologic depth with advancing age [23]. A study successfully and safely treated the condition in children with phototypes IV/V/VI using QS (694 nm, 1064 nm) lasers without anesthesia or sedation, advocating for early intervention to enhance efficacy and avoid psychosocial distress later in life. The study also suggested a trend toward greater clearance with QS ruby in type-IV patients [23]. The ps-QS 755 nm Alexandrite laser has shown superiority over its ns-QS counterpart in terms of fewer treatment sessions required, though PIH was noted in a few cases with darker skin tones [20] (Table 2). Finally, while not formally classified as a dermal nevus, certain subtypes of epidermal nevi may have dermal components (e.g., hair follicles in Becker’s nevus) that need to be addressed in order to reduce the overall appearance of pigmentation in such cases; (refer to authors’ study in Section 3.2 of discussion).

#### 3.1.2. Acquired Dermal Macular Hyperpigmentation (ADMH)

A Delphi consensus designates ADMH as a conglomerate term that comprises disorders clinically characterized by variably sized, pigmented macules and patches with histologic evidence of pigment incontinence [31]. These diseases include erythema dyschromicum perstans/ashy dermatosis, Riehl’s melanosis/pigmented contact dermatitis, lichen planus pigmentosus, and idiopathic eruptive macular pigmentation [32] (Table 1). Careful diagnosis, exclusion of other causes, and monitoring disease evolution for accurate categorization are important considerations. Ensuring disease stabilization prior to initiating laser therapy is important to avoid disease exacerbation, especially for lichen planus pigmentosus [33,34]. Riehl’s melanosis is characterized as an ADMH, manifesting as brownish-grey discoloration on the face and neck. Pigmented contact dermatitis is the term used in cases with a documented history of particular contact allergies [19]. Despite similarities in morphology and histopathology amongst these entities, treatment remains a challenging task with inconclusive outcomes [32]. Non-laser treatment options include topical lightening agents, oral tranexamic acid, glycyrrhizin compound, peels, etc. [35,36]. The most frequently employed is the 1064 nm QS Nd:YAG in the low or mid-fluence range. Numerous studies have explored the use of diverse light/laser therapies, including 1064-nm QS Nd:YAG laser, IPL, 755 nm Alexandrite ps-laser, non-ablative laser, pulsed-type microneedling frequency, etc. (Table 3). However, in practice, the improvement achieved is modest and may not meet the patient’s satisfaction, while treatment with topical bleaching agents alone is unsatisfactory [19,34,37]. Combining both, however, may enhance efficacy [33]. Recently, besides dermal melanin, researchers have identified telangiectatic or dilated vessels in the dermis as potential treatment targets [37]. In an attempt to target both these targets, a recent study combining fractional ps-Alexandrite 755 nm laser and Pulsed Dye laser in a small cohort of patients yielded significant improvement with negligible side effects [37]. (Table 3) Despite efforts, there is a need for further research to characterize these conditions and develop uniformly effective treatments [32].

#### 3.1.3. Tattoos

Tattoos may be decorative, cosmetic, traumatic, medical, or iatrogenic. Decorative tattoos come in amateur and professional forms, with amateur tattoos often in blue-black and easier to remove than denser, multi-colored professional tattoos. The laser wavelength may be selected based on specific pigments/colors [13,16]. Traumatic tattoos resulting from road traffic accidents can often be cleared with one or two treatments, in contrast to decorative tattoos, which often require multiple sessions [13]. Cosmetic tattoos, particularly those used for breast reconstruction surgery or as lip liners, may darken with laser therapy, necessitating test patches and follow-ups. Additionally, caution is advised when treating gunpowder and firework tattoos, as the implanted material may ignite, potentially leading to pox-like scars [13].

QS lasers are the gold standard for tattoo treatment, as they target tattoo ink in the dermis, the density and composition of the tattoo ink influencing the response to laser treatment as well as the number of sessions required [13]. The ink pigments contain both inorganic and organic compounds, and QS lasers are noted to be effective, especially for black, dark blue, and green tattoos, while red and yellow tattoos are relatively challenging to treat. The R20 method, described over a decade ago, involves multiple treatment passes of a QS laser with a 20-min delay between passes within a single session, enhancing tattoo clearance but also epidermal injury and PIH [43]. Since then, significant refinement has occurred in laser treatment methods for tattoo removal. The recent literature extensively documents the safety and efficacy of the ps-laser for treating tattoos in Asian populations [20,44,45,46]. The issue of color dependency in tattoo removal is addressed, and while ps-lasers show less color dependence than ns-lasers, true color independence might only be achieved with even shorter pulse widths [3]. The smaller diameter of a tattoo ink particle, compared to a melanosome, allows for selective photo-thermolysis while safeguarding the surrounding normal tissues [44]. Recommendations based on consensus strength percentages of the German Dermatological Society suggest the use of ps- or ns-ranged QS pigment and vascular (Nd:YAG, Ruby, Alexandrite, KTP) lasers and advise against the use of IPL for all types of tattoos. Ablative laser options (10,600 nm CO_2_, 2940 nm Er:YAG, or Erbium-doped yttrium aluminum garnet) may be considered in specific situations, such as smaller areas not responding to other modalities [16].

A pilot study in a small cohort noted that ps-lasers were more efficient in tattoo removal, requiring fewer sessions (less than half) than ns-QS lasers, with fewer adverse events. The superiority of the ps-Alexandrite 755 nm laser was evident in treating black and multi-colored tattoos, especially for black, blue, and purple colors, and additional sessions with the ps-NdYAG (532 nm) laser were effective for red and yellow colors. Larger prospective comparative trials between ps and ns-domain lasers would be required to strengthen these findings [45]. Combining fractionated and unfractionated 1064 nm ps-laser is more efficacious than the unfractionated laser alone, with superior clearance scores and fewer adverse events, as found in a recent split-tattoo randomized comparative study [47] (Table 4). The mechanism of improvement is proposed to be similar to that of combining a ps-laser with a fractional ablative (e.g., CO_2_) laser, accelerating tattoo clearance and decreasing blistering by allowing the release of tattoo ink and dermal intercellular fluid via microcolumns of epidermal ablation [48].

#### 3.1.4. Post-Inflammatory Hyperpigmentation (PIH)

Inflammatory skin diseases often lead to PIH, a common complication, especially in higher Fitzpatrick phototypes. It often consists of both epidermal and dermal components. While QS lasers and IPL systems can effectively target the epidermal component, the dermal aspect frequently proves resistant to treatment with a risk of worsening pigmentation. Hence, test patches are recommended while using lasers for treatment. Strict sun protection and priming or combined treatment with topical lightening agents are essential [13]. Ps- or ns-ranged QS pigment and vascular (Nd:YAG, Ruby, Alexandrite, KTP) lasers are suggested. The lasers in the short millisecond range (595/585 nm dye, 532 nm KTP, 755 nm Alexandrite) may be considered. Fractional non-ablative (1540–1570 nm Er:Glass, 1927 nm Thulium) lasers and IPL are other potential options. However, ablative (10,600 nm CO_2_, 2940 nm Er:YAG) lasers are not recommended for this purpose. These recommendations are based on consensus strength percentages, and the considerations aim to guide practitioners in choosing suitable laser treatments for PIH, accounting for different laser types and parameters [16,50]. A study treating PIH patients with a fractionated ps-755 nm laser brought 50–75% improvement at 3 years, suggesting that it could serve as a long-term treatment for PIH in Asian skin [51] (Table 5).

#### 3.1.5. Mixed-Type/Dermal Melasma

Strictly compartmentalizing melasma based on pigment levels is challenging, with studies revealing that the majority of cases are, in fact, of the mixed type [53]. Regardless of the treatment modality, the long-term recurrence rates for melasma appear to be high (in contrast to ADMH, nevus of Ota, etc., in which recurrences seldom occur once the lesion has been cleared) [13]. QS-lasers are regarded as the benchmark treatment for treating melasma of all types [3,13]. Due to the multiple sessions needed for laser therapy, adverse events like mottled hypopigmentation may occur, treatable with tacrolimus/targeted phototherapy, but their incidence can also be minimized with specific parameters, including larger spot sizes, reduced fluence, and longer intervals between sessions [3]. Laser toning utilizes repetitive (i.e., multiple passes) sub-threshold (i.e., low fluence) pulses of 1064-nm QS-Nd-YAG laser to treat melasma [3]. By employing the concept of “minimal photothermolysis” with large spot sizes and low energy fluences below the selective photothermolysis threshold (<3 J/cm^2^), melanin pigment undergoes fragmentation and dispersion within the cytosol. This achieves pigment lightening with minimal inflammation or epidermal damage as well as subcellular damage to the upper dermal vascular plexus, addressing one of the implicated pathogenic mechanisms. The improved skin texture is akin to chemical peels, hence the term “laser toning” [3,6]. This is particularly important in melasma, where multiple sessions are often required for successful outcomes, and it may even serve as an alternative for drug-intolerant cases [54]. The use of split-pulse techniques on sensitive skin helps to reduce discomfort [3]. However, success rates vary, and recurrence is high upon laser discontinuation, up to the tune of 81% when applied as monotherapy, highlighting the need for ongoing maintenance and combination treatments for optimizing long-term results [3]. Most studies on melasma utilize the MASI (melasma area severity index) or its modification for objectively assessing and comparing treatment efficacies, and combining laser therapy with other modalities (topical lightening agents, IPL, and fractional 2940-nm Er:YAG laser) appears to enhance this outcome [3]. In a few studies, Pixel Er: YAG has demonstrated significant results for dermal and mixed-type melasma compared to QS-Nd-YAG alone [3,6]. Recent literature focuses on the effectiveness of the fractional ps-domain form of the 1064 nm Nd:YAG [55,56]. In fact, a meta-analysis of six RCTs on ps-lasers for melasma found that the 1064 nm ps-laser significantly reduced MASI with minimal side effects, while the 755 nm ps-laser did not outperform topical agents, leading to PIH. The study suggests the effectiveness and safety of the 1064 nm ps-laser for melasma, with uncertainty regarding other wavelengths [57] (Table 6).

### 3.2. Authors’ Single-Centre Experience of Sequential Laser Therapy in a Cohort of 122 Indian Patients

**Figure 1:**
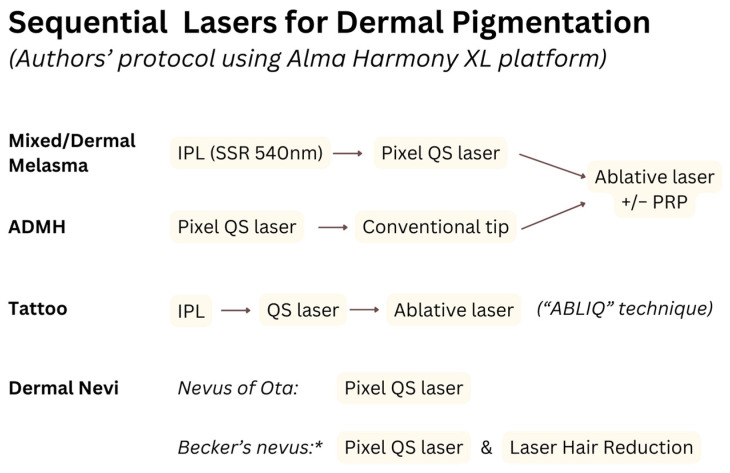
Sequential laser protocol used by authors to treat dermal pigmentation. (* Although not formally classified as a dermal nevus, Becker’s nevus displays ‘darkening’ due to hair follicles extending through the dermis, warranting its discussion here).

Over the years, the authors have tried to assess the efficacy and safety of sequential laser treatments for resistant dermal pigmentation in Indian skin types. Over a five-year period, 122 affected Fitzpatrick skin type III–IV patients were clinically and dermoscopically diagnosed and treated with sequential lasers at the authors’ advanced laser and aesthetic dermatology center, adhering to ethical standards in alignment with the Helsinki declaration. The four groups based on the diagnoses were mixed/dermal melasma, ADMH, dermal nevi, and tattoos. Demographic data, treatment details, and follow-up durations were noted. Various lasers, including ablative pixel Er:YAG, IPL-based technologies, Q-Switched, and “hybrid” lasers (Alma Harmony XL platform), were sequentially used with distinct protocols for each group, and the sessions were performed at 3-weekly intervals. The rationales of the laser combinations were based on their specific mechanisms. (Table 7, Figure 1) Standard clinic protocol involved ensuring patient consent, screening contraindications, and correcting nutritional deficiencies guided by blood investigations, with an emphasis on regulating meal timings [63,64]. Post-procedure, the use of antibiotic-topical steroid cream for two days was advised, in addition to sunscreen and ample emollient application. Follow-up recommendations included non-hydroquinone-based topical treatments for melasma patients (Kojic acid cream in the evening and a retinol-based preparation at night), continued sunscreen application, and sun protection. Baseline photographs were compared with intervals to assess relapse, and the mean treatment duration ranged from 5–9 months.

**Figure 2:**
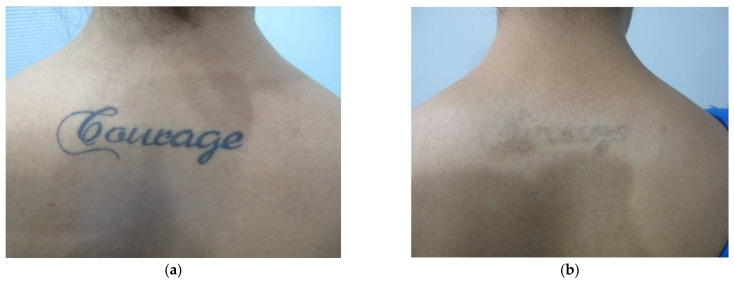
(**a**) Tattoo on back—before treatment (**b**) Tattoo on back—after treatment.

**Figure 3:**
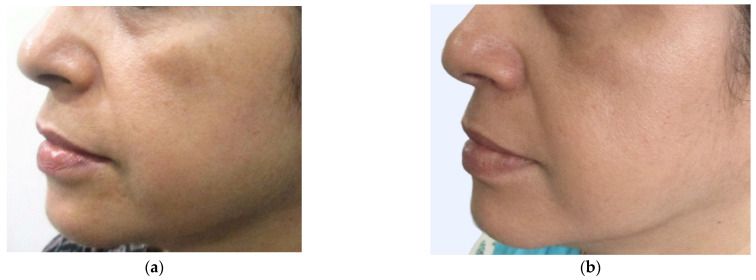
(**a**) Melasma patch in the malar area—before treatment (**b**) Melasma patch in the malar area—after treatment.

**Figure 4:**
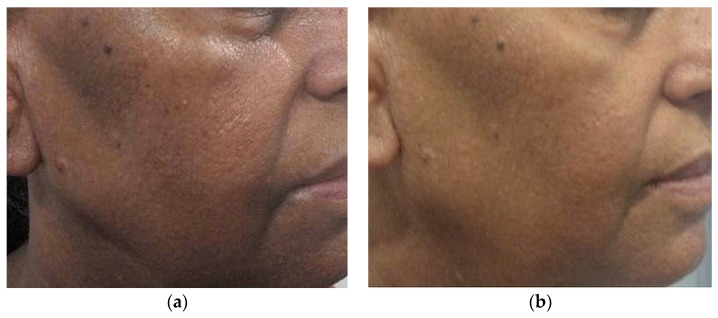
(**a**) Lichen planus pigmentosus—before treatment (**b**) Lichen planus pigmentosus–after treatment.

**Figure 5:**
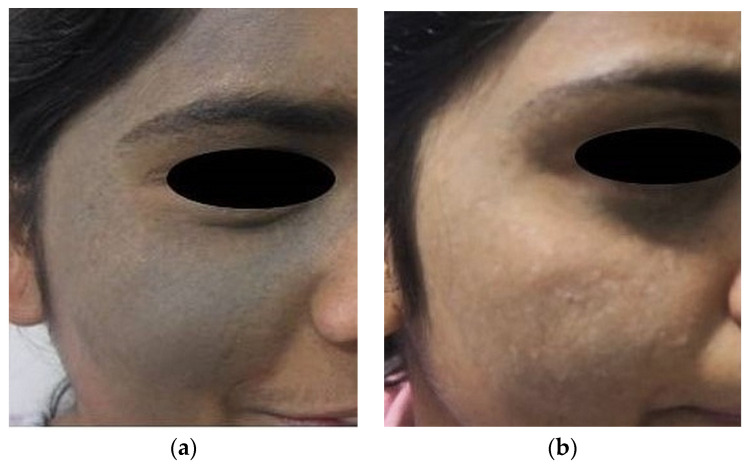
(**a**) Nevus of Ota—before treatment (**b**) Nevus of Ota—after treatment.

Physicians subjectively assessed the response to therapy using an improvement scale (1 to 5) based on photographs, where a higher score indicated greater improvement, while patient feedback was obtained through a Likert scale ranging from 0 to 10. (Table 8) The treatment, spanning 4–10 sessions at three-week intervals, demonstrated a substantial reduction in pigmentation for all groups (Table 8, Figure 2, Figure 3, Figure 4 and Figure 5). The study results underwent statistical analysis using Stats-kingdom (Supplementary Data excel sheet). In examining the effectiveness of laser therapy across different pigmentary conditions, descriptive statistics of physician assessment scores, ranging from 1 to 5, revealed varying means ± SD as follows: Nevi (3.76 ± 1.30), Tattoo (4.46 ± 0.71), ADMH (3.81 ± 1.33), and Melasma (3.84 ± 1.07). The one-way ANOVA indicated a statistically significant difference in mean improvement scores amongst the various pigmentary conditions (F = 3.39, *p* = 0.02). Further analysis using Tukey’s HSD test to assess pairwise distinctions amongst the groups revealed that each pigmentary condition responded uniquely, with melasma patients showing a significantly higher mean improvement score compared to tattoos (*p* = 0.03). This highlights the importance of considering the specific pigmentary condition while planning treatment, patient counseling, and evaluating laser treatment efficacy. Customization of laser therapy strategies based on the nature of pigmentation is important to optimize treatment outcomes for patients with diverse dermal pigmentary conditions.

While the authors inferred that sequential laser therapy in the given protocols, along with conventional topical therapies, is safe and effective for treating dermal pigmentation in Indian skin, incorporating objective methods for assessing improvement in pigmentation would have been meaningful, allowing an assessment of statistical changes in the level of pigmentation within each group and, thereby, a more precise evaluation of the effectiveness of each group’s protocol. Also, it is acknowledged that the availability of specific laser types to perform the mentioned protocols may be a limiting factor. While the use of multiple lasers may be perceived as a challenge, the study’s protocols allow flexibility. Centers may customize and adapt the suggested approach based on their available technologies and expertise, understanding the key principles behind the combination of lasers and tailoring the treatment accordingly. This study can serve as a catalyst for further research to explore simplified or alternative laser combinations that may be more accessible to a broader range of clinics. The use of a sequential combination of lasers in the protocols is a strength rather than a limitation, as it reflects a thoughtful and individualized approach to dermal pigmentation treatment. Additionally, correcting various macro and micro-nutrient deficiencies and incorporating a protein-rich diet in the early morning was suggested to promote healing, potentially reducing PIH based on the authors’ previous experience [63,64]. Further research could be conducted to validate these findings.

There is limited literature on sequential lasers in treating dermal pigmentation. A study investigating the impact of sequentially delivering long-pulsed 755 nm Alexandrite and 1064 nm Nd:YAG laser pulses revealed their ability to eliminate pigment chromophores in tissue-mimicking phantoms with black tattoo ink, suggesting potential efficacy for tattoo removal [65]. Sequentially combining unfractionated and fractionated 1064 nm ps-lasers yielded superior tattoo clearance and fewer adverse events than using the former alone [47]. Nam et al. studied the nevus of Ota patients receiving a combination of lasers in different settings [30]. Sequentially combining fractional alexandrite ps-laser and pulsed dye laser improved the treatment effectiveness for Riehl’s melanosis through dual action [37] (Table 3 and Table 4). The author’s firsthand experience offers insights into the application of various sequential lasers based on their mechanisms, urging further research to validate and expand upon these findings.

### 3.3. Minimizing Adverse Effects

The review attempts to identify various laser systems and strategies for minimizing adverse effects in darker skin tones. Despite advancements, complications such as PIH, mottled hypopigmentation, purpura, etc., have been described with laser treatment of dermal pigmentation, especially considering the higher number of sessions required compared to epidermal lesions [6,13]. Mitigating these risks involves meticulous attention to parameters like spot sizes (should be larger) and intervals between sessions (should be longer) [6]. Unlike treating epidermal lesions, where the spot size should cover just the lesion to avoid affecting surrounding skin, dermal lesions require a spot size that results in immediate whitening (“frosting”) as an endpoint to achieve deeper penetration and reduced tissue splatter [13]. The recommended interval between treatment sessions is six to eight weeks. Longer intervals, up to six months, are suggested for treating nevus of Ota [13]. Broadly speaking, starting with the lowest fluence that elicits a visible response is always prudent, adjusting if the initial outcome is suboptimal [13]. Specific approaches, such as laser toning, fractionated beam, short PD (picosecond technology), split-pulse technique, avoiding excessive overlap during treatment, and implementing cooling measures such as continuous air cooling may be employed to achieve pigment lightening without causing significant damage to the epidermis [3,6]. Laser toning is a subcellular selective photo-thermolysis approach that minimizes inflammation, involving weekly sessions and parameters like low-fluence, high peak power, ultrashort PD, multiple passes, and a flat-top beam to selectively destroy melanin without inducing cell death [3,6]. Combination therapies with suitable topical modalities for ongoing maintenance, sequential laser therapy, and implementing precautions in darker skin phototypes, including sun protection, priming with skin-lightening agents, conducting test patches, and customizing parameters according to manufacturer specifications enhance treatment outcomes while minimizing potential damage. Patient counseling for molding realistic expectations prior to initiating the procedure is important [6,13,16]. Postoperative care, including ice pack application and regular use of broad-spectrum sunscreens, is advisable [13].

### 3.4. Literature Gap and Future Directions

More inclusive, large-scale studies and randomized controlled trials (RCTs) that consider the heterogeneity within SoC would be relevant to provide evidence regarding the effectiveness of recent and innovative treatments within such populations. Incorporating extended follow-up durations would help draw meaningful conclusions since the reduction in dermal pigmentation is slow, with a potential for relapse. For conditions like ADMH that have a variable and unpredictable course with a potential for spontaneous resolution, it would be helpful to include a control group to evaluate whether the observed improvement may be ascribed to the treatment method [12]. The use of the 1064 nm ps-laser in ADMH is yet to be explored; additional research is needed to characterize this condition and establish consistently effective treatments [32]. For tattoos, larger prospective comparative trials on ps- and ns-domain lasers are lacking [45]. Lastly, there exists notable heterogeneity in the laser types, treatment settings and disease severity assessment methods employed. Future explorations into laser technologies may focus on defining criteria for laser selection and standardizing processes such as patient selection, laser protocols, and adopting objective methods for evaluating clinical improvement. At the same time, it is essential to recognize that certain parameters require individualization to achieve optimal outcomes. Given the link between specific micro-/macro- nutrient deficiencies, meal timings, etc., with various cosmetic dermatological conditions, including PIH, future studies could pursue an investigation involving pre- and post-treatment blood tests to address diverse deficiencies, implementing appropriate laser protocols and dietary modifications to explore a potential correlation between nutrition and pigmentation [63,64].

## 4. Conclusions

The review provides a comprehensive and detailed overview of the treatment landscape for dermal pigmentation in SoC, covering a spectrum of conditions and offering insights into both challenges and promising therapeutic approaches. The underlying laser–tissue interactions, consensus recommendations, and therapeutic potential in cases resistant to conventional modalities have been highlighted. Specific laser technologies and their applications have been covered, underlining the ongoing quest for optimal protocols, safety, and efficacy in dermatological laser treatments. The insights gained from the authors’ experience in a cohort of 122 Indian patients further validate the use of sequential laser therapy in treating dermal pigmentation in SoC, offering a direction for future research and clinical practice.

## Figures and Tables

**Table 1:** Commonly encountered disorders of dermal and mixed hyperpigmentation.

Dermal Hyperpigmentation	Acquired Dermal Macular Hyperpigmentation Lichen Planus Pigmentosus Erythema dyschromica perstans Ashy dermatosis Idiopathic eruptive macular pigmentation
	Pigmented Contact Dermatitis, Riehl’s melanosis
	Dermal Melanocytosis
	Congenital Dermal Melanocytosis
	Nevus of Ota, Nevus of Ito, Hori’s Nevus, Sun’s Nevus
Mixed Epidermal–Dermal Hyperpigmentation	Post-inflammatory Hyperpigmentation (PIH)
	Melasma
	Drug-Induced Hyperpigmentation

**Table 2:** Summary of advanced treatments for dermal melanocytosis (nevus of Ota and Hori’s nevus) in SoC based on recent studies.

Author	Study Type	Year	Laser	Therapy Parameters	Fitzpatrick Scale	No. of Cases	Clinical Outcome	Adverse Events
Imagawa et al. [24]	Prospective comparative study	2023	550-ps 755 nm and 50-ns 755 nm lasers	Clinical endpoint for fluence choice was immediate whitening, with fluence ranging from 2.33 to 3.36 J/cm^2^ for ps laser and 5.5 to 7 J/cm^2^ for ns laser. PD: 550 ps and 50 ns for respective lasers; 2.5–3 mm spot	III–IV	10	Ps laser had superior efficacy, requiring fewer average sessions (4.2) than ns-laser (5.4)	Hyper- and hypopigmentation occurred in the ns group. The ps group had no side effects.
Yang et al. [25]	Retrospective study	2022	ps-755 nm Alexandrite laser	Fluence 1.96–2.08 J/cm^2^, 3.5–4.0-mm spot	III-IV	86	96.5% of patients achieved >95% clearance in an average of 4.3 sessions. Early onset of lesions (<5 months of age) and darker skin types (type IV vs. III) significantly increased the number of sessions required for clearance. Age at first treatment, sex, and nevus color had no significant effect.	Transient hyper- and hypopigmentation
Koh et al. [20]	Retrospective review	2020	Picosecond 755-nm laser	For NO, the average fluence was 2.02 J/cm^2^, and for HN, it was 2.08 J/cm^2^	III/IV	29	In the NO group, mean pre-and-post-treatment Physician global assessment scores were 3.1 and 1.3, respectively (1.8-point change, *p* = 0.0002). In HN group, mean pre-and-post-treatment PGA scores were 2.6 and 1.1, respectively (1.5-pt change, *p* = 0.004)	Eleven patients (37.9%) experienced post-laser erythema, and 1 (3.4%) developed transient post-laser hypopigmentation.
Ge et al. [27]	RCT (Split-lesion study in nevus of Ota)	2020	Ps-755-nm Alexandrite laser versus ns-QS 755-nm Alexandrite laser	Each lesion was treated with single-pass method in up to 6 sessions at 12-week intervals. ps-laser: 2–4 mm spot, 1.59–6.37 J/cm^2^, 5 Hz. Ns-laser: 3 mm spot, 5–7 J/cm^2^, 5 Hz.	III–IV	56	Higher efficacy, decreased pain scores, and post-inflammatory hyper/hypopigmentation in the ps laser-treated group.	There was an improved clearance, with fewer side effects and more patient satisfaction in the ps-laser arm.
Hu et al. [28]	Retrospective study	2021	Ps-755-nm Alexandrite laser	Fluence 2.73–3.98 J/cm^2^, spot size 2.9 to 2.4 mm, PD 650-ps, 1 to 4 sessions.	III–IV	36	88.89% of patients had moderate to marked improvement.	Transient swelling and erythema. Transient PIH (in two patients) and hypopigmentation (in one patient) that resolved in 6 weeks.
Yu et al. [29]	RCT (Split-face study in Hori’s nevus)	2018	ps 755-nm Alexandrite laser vs. ns-QS 755-nm Alexandrite	12-week interval. ps-laser: 2–2.5 mm spot, 4.07–6.37 J/cm^2^, 2.5 Hz. Ns-laser: 3 mm spot, 6–8 J/cm^2^, 2 Hz.	III–IV	30	The PSAL-treated area achieved significantly better clearance (3.73 vs. 2.4) with less severe pain (4.47 vs. 5.16)	the PSAL and QSAL treatments, lasting for nearly one and a half months
Belkin et al. [23]	Retrospective case series	2018	QS lasers (Ruby 694 nm in type IV and QS Nd:YAG 1064 nm in types V, VI)	Patients were treated without general anesthesia or sedation; corneal shields were used where appropriate.	IV–VI	24 (children <18yrs)	Excellent response (76–100% improvement) in 70% of patients and good to excellent response (51–100% improvement) in 86%. Fewer sessions/lesser fluence required and fewer complications in younger patients.	Two patients (8%) had post-inflammatory hyperpigmentation, one of whom also had focal hypopigmentation.
Nam et al. [30]	Retrospective study	2017	Multiple QS laser modalities	Settings varied for different laser machines.	/	67	An average of 19 sessions (range 10–27, *p* = 0.001) is required for 95% clearance.	Two patients (3%) had persistent side effects, e.g., atrophic scarring, though not mentioned how many sessions these patients underwent.

PD Pulse Duration; NO Nevus of Ota; HN Hori’s Nevus; / refers to detail not mentioned in study.

**Table 3:** Summary of advanced treatments for acquired dermal macular hyperpigmentation (lichen planus pigmentosus, Riehl’s melanosis, etc.) in SoC based on recent studies.

Author	Study Type	Year	Laser	Therapy Parameters	Fitzpatrick Scale	No. of Cases	Clinical Outcome	Adverse Events
Kim et al. [37]	Open-label, Mixed-methods study	2023	Combination of fractional alexandrite picosecond laser and PDL	3 to 7 sessions 4 weekly intervals. For ps-laser, single treatments, 2–3 passes, <10% overlap, ≥3000 pulses to full face, fluence 0.25 J/cm^2^, PD 750 ps, 10 mm spot, frequency 10 Hz. For PDL, fluence 7 J/cm^2^, PD of 6ms, 7 mm spot	III–IV	11	Significant reduction in assessment scores in all patients—10 out of 11 reported being very satisfied or satisfied. Effective for both pigmentation and dilated vessels	Transient erythema and oedema
Kim et al. [38]	Retrospective Study	2021	Non-ablative 1927 nm Fractional Thulium Fiber Laser	5 W for the output power and 10–20 mJ pulse energy at monthly intervals	III–IV	9	6/9 had “marked”, 1/9 had “significant” and 2/9 had “moderate” improvement	Mild erythema, which faded away within 3 days
Bhari et al. [39]	Prospective, pilot study	2020	QS Nd:YAG 1064 nm laser	QS-NdYAG laser, toning protocol (3 J/cm^2^/6 mm/10 Hz), 6 sessions, 2 weekly intervals	IV–V	9	25.7% improvement as per physician assessment. Moreover, 8/9 did not perceive any improvement. No change in melanin index or erythema index	Post-inflammatory hypopigmentation in one patient
Cho et al. [40]	Retrospective Study	2020	Mid-fluence QS Nd:YAG 1064-nm laser	3.5–5 J/cm^2^, 5-mm spot, 10 Hz, 6 sessions, at 5-week interval	III–IV	21	6/21 had “moderate”, 8/21 had “much” and 2/21 had “very much” improvement”	No severe side effect
Shah et al. [41]	Open-label, non-randomized prospective pilot study	2019	QS Nd-YAG 1064 nm laser	5 sessions, 4–8 weekly, 5-mm spot, fluence 3–4.6 J/cm^2^, 5 Hz, 5-mm, single pass with minimal overlap	IV–V	13	38.4% had >90%, 38.4% had >75%, and 23% had >50% improvement	Confetti-like depigmentation in one patient, mild scarring in the supraorbital area in one patient
Choi et al. [42]	Retrospective Study	2019	1064-nm Nd:YAG laser	Fluence 0.9 J/cm^2^ to 2.0 J/cm^2^, 7 mm spot, 10 Hz, 3-week intervals with 4% HQ cream	III–IV	10	7/10 “near total improvement” 2/10 “marked improvement” 1/10 “minimal improvement	Three patients complained of guttate hypopigmentation
Kwon et al. [33]	Pilot study	2017	Low-fluence Q-switched 1064-nm Nd:YAG, combined with other lightening agents.	Fluence 1–1.3 J/cm^2^, 10 mm spot, 2–3 passes, at 3-week intervals, 4% HQ cream and oral tranexamic acid 250mg/day	III–V	8	3/8 “almost clear” grade 5/8“marked improvement” grading	None

**Table 4:** Summary of advanced treatments for tattoos in SoC based on recent studies.

Author	Study Type	Year	Laser	Therapy Parameters	Fitzpatrick Scale	No. of Cases	Clinical Outcome	Adverse Events
Sirithanabadeekul et al. [47]	RCT	2022	Fractionated 1064-nm picosecond lasers and unfractional 1064-nm ps-laser	1064-nm ps-laser: fluence 1.5–7.24 J/cm^2^; 3–4.5 mm spot, 2–5 Hz; fractionated 1064-nm ps-lasers: fluence 0.8 J/cm^2^; 8 mm spot, 2–5 Hz	III–V	11	The combination side showed greater clearance scores and fewer adverse events than the side of unfractional 1064-nm picosecond laser alone.	Temporary crusting, purpura, edema, erythema, burning sensation, and petechiae
Kasai et al. [45]	Retrospective case series	2017	Multi-colored tattoos with a ps 755 nm Alexandrite laser and a ps Nd:YAG laser	/	/	4	Ps-lasers require fewer sessions than ns-QS lasers for treating black-colored tattoos and are more efficient than ns-QS lasers for multi-colored tattoos.	Transient PIH in 1 patient
Kauvar et al. [49]	Prospective clinical study	2017	Dual-wavelength, 1064/532-nm, ps-laser	Both wavelengths are used on the same tattoo. Spot sizes 2 to 6 mm, maximum fluence 1.9 J/cm^2^ for 532-nm and 8.5 J/cm^2^ for 1064-nm handpieces	Only 26% of subjects were non-Caucasian	34 (34 subjects with 39 tattoos)	86% (31 of 36 tattoos) showed at least a 50% clearance after three treatments. Patient satisfaction and treatment tolerability were high.	Adverse events were few and transient in nature.
Sirithanabadeekul et al. [47]	RCT	2022	Fractionated 1064-nm picosecond lasers and unfractional 1064-nm ps-laser	1064-nm ps-laser: fluence 1.5–7.24 J/cm^2^; 3–4.5 mm spot, 2–5 Hz; fractionated 1064-nm ps-lasers: fluence 0.8 J/cm^2^; 8 mm spot, 2–5 Hz	III–V	11	The combination side showed greater clearance scores and fewer adverse events than the side of unfractional 1064-nm picosecond laser alone.	Temporary crusting, purpura, edema, erythema, burning sensation, and petechiae

/ Refers to details not mentioned in study.

**Table 5:** Summary of advanced treatments for post-inflammatory hyperpigmentation in SoC based on recent studies.

Author	Study Type	Year	Laser	Therapy Parameters	Fitzpatrick Scale	No. of Cases	Clinical Outcome	Adverse Events
Bae et al. [52]	Retrospective photographic analysis	2020	Non-ablative fractional 1927 nm laser	Low energy, low-density parameters (fluence 5 mJ, spot size 140 μm, depth 170 μm, 5% coverage)	IV–VI	61	A mean improvement of 43.24% was assessed by two independent dermatologists, and there was a statistically significant correlation between the raters.	No side effects reported.
Li et al. [50]	Prospective interventional study	2018	Intense pulsed light	Wavelength 560–615 nm, 2–6 treatments over 3–5 weeks	III–IV	35	IPL significantly lessened PIH as per physician assessment with an 82.9% satisfaction rate	No post-treatment complications
Kasai et al. [45]	Retrospective case series	2017	Fractionated 755 nm Alexandrite ps-laser	8 mm spot, fluence 0.4 J/cm^2^, 750-ps, 10 Hz, 3 sessions at 1- or 2-month intervals	III	1	At the 3-year follow-up, the PIH lesions had a 50–75% improvement	Deepening of local skin lesions

**Table 6:** Summary of advanced treatments for melasma in SoC based on recent studies.

Author	Study Type	Year	Laser	Therapy Parameters	Fitzpatrick Scale	No. of Cases	Clinical Outcome	Adverse Events
Manuskiatti et al. [58]	RCT	2022	755 nm fractionated and un-fractionated ps-laser	Fractionated on one side of the face and flat optics (unfractionated)on the other side: 8-mm spot, fluence 0.40 J/cm^2^, 2.5 Hz, 5 sessions at 5-month intervals	IV–V	18 (14 patients completed)	All patients showed significant improvement in pigment clearance without notable differences between treatment areas.	Mild PIH and erythema
Hong et al. [59]	RCT	2022	Ps Nd:YAG vs. conventional 1064 nm Nd:YAG	10-mm spot, fluence 1.5–2.5 J/cm^2^	III–IV	19	Both arms had comparable outcomes in terms of modified MASI and melanin index	Mild pain
Wong et al. [56]	Prospective study	2021	Fractional 1064-nm ps-laser	450-ps pulses with maximum microbeam energy per pulse 3 mJ	III–IV	10	Compared to baseline, seven patients had moderate to marked improvement 6 weeks post-therapy, and five had sustained improvement.	Erythema, edema, and hyper/hypopigmentation
Wang et al. [60]	RCT	2020	Fractionated ps-Alexandrite 755 nm laser versus TCC	8 mm spot, fluence 0.4 J/cm^2^	/ (Asian ethnicity)	29	Fractionated ps-Alexandrite laser proved as effective as TCC for treating melasma.	Dryness, PIH Itching focal desquamation
Chen et al. [61]	Prospective study	2019	Fractionated 755 nm ps-Alexandrite laser	8-mm spot, fluence 0.4 J/cm^2^, 750 ps for 3 treatment sessions at 4-to-6-week intervals	IV	20	The mean MASI score improved to 6.9 ± 3.7 after 3 sessions of treatment, with the baseline being 9.4 ± 4.7	Erythema, pruritus, scaling, one developed PIH.
Garg et al. [6]	RCT	2019	SSR 540 nm, low fluence pixel-QS Nd:YAG, and pixel-Er: YAG	SSR: 8–9 J/cm^2^, one pass of double shots; pixel-QS Nd:YAG 1064-nm: 4 Hz, low fluence 1.2 J/cm^2^, 4 passes; pixel Er:YAG 2940 nm: 1100–1200 mJ/pass, long pulse, stack mode, 4–5 passes	III–IV	60	All groups had a significant reduction in mMASI (*p* < 0.001). SSR and pixel-QS Nd:YAG worked best on epidermal melasma (*p* < 0.001), while pixel-Er: YAG laser was most effective for dermal and mixed melasma (*p* < 0.001).	Transient and treatable side effects (PIH, acneiform eruptions. One patient in pixel-Er: YAG developed herpes labialis, treated with Valaciclovir
Chalermchai et al. [55]	RCT	2018	Fractional picosecond 1064-nm laser and 4% HQ	Fractional ps-1064-nm laser: fluence 1.3–1.5 mJ/microbeam, PD 450 ps, 4 Hz versus daily 4% HQ cream	III–IV	30	The intervention sides had considerably lower melasma area severity index scores than 4% HQ cream alone.	Mild erythema, desquamation, burning sensation
Choi et al. [62]	RCT	2017	Dual wavelength ps-laser 1064 and 595 nm with 2% HQ, vs. 2% HQ alone	7–10 mm spot, fluence 0.2–1.5 J/cm^2^/5 mm spot, and fluence 0.1–0.55 J/cm^2^	III–IV	39	Ps-laser with 2% HQ outperformed 2% HQ alone; satisfaction scores supported the results.	Mild dermatitis

PD Pulse Duration; MASI Melasma Assessment Severity Index; TCC hydroquinone 4% + fluocinolone acetonide 0.01% + tretinoin 0.05%; HQ hydroquinone; / refers to detail not mentioned in study.

**Table 7:** Clinico-demographic profile, laser protocols, and treatment details of affected patients.

Diagnosis	No. of Patients	Age in Years (Mean ± SD)	Laser Protocols (Alma Harmony XL Platform)	Rationale behind Laser Sequence	No. of Sessions (Mean ± SD)	Follow-Up in Months (Mean ± SD)
Mixed/dermal Melasma	51	44.5 ± 8.2	Sessions 1, 2: Non-ablative laser (SSR 540), 7–8 J/cm^2^, 30 Hz Sessions 3, 4: Pixel QS 1064 nm, 1200 mJ/cm^2^, 4–8 passes 5th session: Ablative (pixel Er: YAG 2940 nm) laser, 1100–1200 mJ/pass, stack mode, long pulse, 4–5 passes Residual areas: “Hybrid” laser (Alma) with CO_2_ and 1570 nm Erb glass fiber wavelengths	Targets various mechanisms of melasma, especially in resistant cases	8.3 ± 2.2	10.4 ± 3.5
Dermal Nevi	21	19.7 ± 4.5	Nevus of Ota, Hori’s nevus: Pixel QS 1064 nm laser for 4–6 sessions starting with 1200 mj, 20 passes, with gradual increase to 40–50 passes. After ≥3 sessions for resistant areas, conventional 3- or 5-mm tips were used at 1000–1200 mJ/pass; single/double passes. Becker’s nevus *: Hair removal: Diode laser (10 J/cm^2^ × 4 passes) → Alexandrite laser (13 J/cm^2^ × 10 passes) for finer hair in subsequent sessions. Pigment reduction: QS-Nd:YAG laser (5–6 sessions) using a 5- or 3-mm tip with 1000 mJ/pass X 1–2 passes.	Lasers customized to address different depths and components of Nevi. For instance, treating Becker’s nevus involved sequential use of hair removal and pigment lasers.	6.4 ± 1.9	8.6 ± 2.1
ADMH	11	21.4 ± 5.2	Sessions 1–3: Pixel QS lasers, 1200 mJ, 4–6 passes Sessions 4, 5: QS 1064 nm laser, 1000 mJ, spot size 3–5 mm Sessions 6, 7: Ablative (pixel Er:YAG) laser, 1200 mJ/pass, 5 passes, long pulse Resistant areas: “Hybrid” laser comprising of CO_2_ and 1570 nm Erbium glass fiber lasers	Serial use of different wavelengths targets chromophores at varying depths.	5.9 ± 1.4	9.1 ± 2.1
Tattoo	39	28.5 ± 4.2	Total of 6 to 7 sessions of the “ABLIQ” technique. This is a single-session cocktail laser therapy that stands for Ablative laser, IPL, and QS laser. But the sequence to be used is as follows: IPL → QS Nd:YAG laser → Ablative lasers viz. either pixel Er:YAG or fractional CO_2_ lasers. (Figure 1)	Facilitates pigment extrusion, reduction in the number of sessions, and “ghost effect” (residual faint outline)	5.9 ± 1.4	9.4 ± 3.1

* Footnotes: While formally categorized as an epidermal nevus, Becker’s nevus exhibits “darkening” influenced by hair follicles extending through the dermis, justifying its inclusion in this discussion.

**Table 8:** Subjective improvement in pigmentation at 6 months follow-up, compared to baseline, based on patient feedback (Likert scale) and physician assessment (photographic analysis).

Type of Dermal Pigmentation (Number of Patients)	Improvement in Pigmentation at 6 Months Post-Therapy, Compared to Baseline					
	Patient Feedback	Physician Assessment				
	Likert Scale (0 to 10)	Excellent (>90% Improvement; Score: 5)	Good (75–90% Improvement; Score: 4)	Moderate (50–74% Improvement; Score: 3)	Mild (25–49% Improvement; Score: 2)	None (<25% Improvement; Score: 1)
Mixed/dermal Melasma (51)	6.9 ± 2.5	18 (35.3%)	13 (25.4%)	15 (29.4%)	4 (7.8%)	1 (1.9%)
Dermal Nevus (21)	6.2 ± 1.4	8 (38%)	5 (23.8%)	5 (23.8%)	2 (9.5%)	1 (4.7%)
ADMH (11)	5.9 ± 1.4	4 (36.3%)	4 (36.3%)	1 (9%)	1 (9%)	1 (9%)
Tattoo (39)	7.9 ± 1.1	23 (58.9%)	11 (28.3%)	5 (12.8%)	0	0

## Data Availability

The data presented in this study are available on request from the corresponding author.

## Supplementary Materials

The following supporting information can be downloaded at: https://www.mdpi.com/article/10.3390/jcm13072116/s1, Data excel sheet.
